# Patients’ and Therapists’ Experiences of Standardized Group Cognitive Behavioral Therapy: Needs for a Personalized Approach

**DOI:** 10.1007/s10488-023-01301-x

**Published:** 2023-09-23

**Authors:** Jasmin Rejaye Gryesten, Stig Poulsen, Christian Moltu, Elisabeth Belmudez Biering, Kirsten Møller, Sidse Marie Arnfred

**Affiliations:** 1 Psychiatric Research Unit, Copenhagen University Hospital – Psychiatry Region Zealand, Slagelse, Denmark; 2 Department of Clinical Medicine, University of Copenhagen, Copenhagen, Denmark; 3 Department of Psychology, Faculty of Social Sciences, University of Copenhagen, Copenhagen, Denmark; 4 Department of Psychiatry, District General Hospital of Førde, Førde, Norway; 5 Department of Health and Caring sciences, Western Norway University of Applied Science, Førde, Norway; 6 Mental Health Centre Ballerup, Mental Health Services of the Capital Region of Denmark, Ballerup, Denmark

**Keywords:** Depression, Personalizing psychotherapy, Group psychotherapy, Psychiatry, Routine Outcome Monitoring, Thematic Analysis

## Abstract

Personalizing psychotherapy can be challenging within standardized group Cognitive Behavioral Therapy (CBT), in which sessions are structured according to a protocol and must accommodate the needs and preferences of multiple patients. In the current study, we aimed to examine patients’ and therapists’ experiences of standardized group CBT and identify their perceptions of different patient needs. Furthermore, we explored how these needs can inform possible content of add-on interventions for patients who are not improving as expected during group CBT.

We conducted 21 individual in-depth interviews with patients with depression and their therapists about their experiences during group CBT with Routine Outcome Monitoring (ROM) and feedback. Interviews were analyzed by using a hermeneutic-phenomenological thematic analysis. Five themes, representing different patient needs, were identified: (1) Individual attention, (2) Psychological exploration, (3) A focus on the patient’s life outside of therapy, (4) Extended assessment, and (5) Agreement on therapeutic tasks.

The study supports that “one size does not fit all” when it comes to psychotherapy. Patients have varying needs when they are not making progress in therapy, and these needs, when unmet, can negatively impact the overall experience of group CBT. By acknowledging the unique needs of each patient and providing additional individual sessions as necessary, we can move towards a more personalized approach that maximizes the benefits of group psychotherapy.

**Supplementary Information:**

The online version contains supplementary material available at 10.1007/s10488-023-01301-x.

## Introduction

It has been demonstrated that group psychotherapy is equally effective compared with individual psychotherapy, with approximately 50% of patients experiencing remission following treatment (Burlingame et al., 2013; Cuijpers et al., 2011, 2014). Group psychotherapy offers several advantages, including that patients can feel validated by other group members, observe and learn from one another, and receive social support within the group setting (Burlingame et al., 2001; Kivlighan & Kivlighan Jr, 2016; Whitfield, 2010). While group Cognitive Behavioral Therapy (CBT) has shown the same effectiveness as individual CBT (Cuijpers et al., 2008), and CBT is a recommended treatment for depression (National Institute for Health Clinical Excellence, 2004), there are limitations to the group format. Previous research has identified issues such as patients’ reluctance to discuss personal problems within the group, a need for additional individual sessions, and a belief among therapists that many mental health patients require more intensive, individualized interventions (Bryde Christensen et al., 2022; Whitfield, 2010). Furthermore, the inflexibility of standardized group CBT has been acknowledged as a problem (Bryde Christensen et al., 2022), and there is a call for the incorporation of more personalized treatment within the framework of group CBT and other standardized formats (Stiles et al., 1998; Whitfield, 2010).

Routine Outcome Monitoring (ROM) with feedback in psychotherapy can potentially reduce adverse outcomes by providing patients and therapists with insight into progress and serving as a guide for adjusting therapy to individual patient needs and preferences (Barber & Resnick, 2022; Bickman, 2008). In ROM, data from quantitative questionnaires are applied to keep track of progress. In cases where patients are not making the expected progress during therapy, the term “Not On Track” (NOT) is applied (Boswell et al., 2015). ROM has the potential to facilitate shared decision-making (Brooks Holliday et al., 2021) and keep sessions goal-oriented (Moltu et al., 2018). However, ROM has shown less promising results in group psychotherapy than individual psychotherapy (Davidsen et al., 2017; Koementas-de Vos et al., 2018; Tasca et al., 2019). One of the reasons for this might be the difficulties in personalizing treatment to the individual in a group context. In group psychotherapy, and especially in manual-based group psychotherapy, personalizing therapy to suit one group member’s needs might be in conflict with the needs and preferences of other group members. Therefore, personalization within groups has to be implemented within the limits of the overall group dynamics. Alternatively, personalization can be implemented outside of the group context, in a format where the therapist has a supplementary discussion with the patient, either informally or as part of the treatment program. Both ways of personalizing differ from individual therapy, where personalization can unfold continuously during therapy. We will argue that there is a lack of coherent operationalization of personalization within group CBT and that such personalization should be informed by patients’ and therapists’ experiences.

Personalization in psychotherapy focuses on what works for specific patients under specific circumstances (Norcross & Cooper, 2021). Studies have explored matching patient characteristics with specific treatments (Cuijpers et al., 2016), matching patient preferences with treatment (Swift et al., 2018), and using patient feedback to adjust the treatment (de Jong et al., 2021). Studies of patterns of change have demonstrated that patients with depression respond differently to different types of therapy. For instance, in a study of patterns of change for patients with depression attending group CBT and individual therapy, Moggia et al. (2020) found that a group of patients needed individual therapy after group therapy in order to improve. The study also demonstrates that high levels of depression symptoms and psychological distress are associated with a higher likelihood that the patients profit from individual therapy rather than group therapy. Furthermore, the greater the patients’ impairment the less likely they were to not benefit from either group or individual therapy. The exploration of patients’ and therapists’ experiences and patient needs during group CBT provides information that is highly relevant to efforts to examine how we can adjust therapy for those patients that do not profit from group CBT for depression. Therefore, and because of the paucity of empirical research into patients’ and therapists’ experiences in personalizing group CBT, we aimed to investigate the research question: What are patients’ and therapists’ perceptions of patients’ needs during standardized group CBT? Moreover, we aimed to study how patients’ and therapists’ experiences could inform relevant content for individual add-on interventions to be applied when patients are not improving as expected during group CBT.

The study is part of a research project that aims to develop content for individual add-on interventions to group CBT and later evaluate the feasibility and effectiveness. The current study serves as a ‘needs assessment’ (Guetterman & Fetters, 2018) that will inform future studies. The term ‘needs’ refers to the informants’ wishes or preferences in the context of group CBT and should not be confused with more basic human needs.

## Method

### Setting

The Danish Mental Health Services are publicly financed and structured as time-limited standardized treatment programs. The treatment for depression is fixed to include a maximum of 18 h of clinicians’ time, which includes assessments, psychotherapy, and medical consultations (Danske Regioner, 2017). Most outpatient clinics primarily offer group CBT as psychotherapeutic treatment for patients with depression with the alternative being 5–7 individual CBT sessions.

The study took place in an outpatient psychiatric clinic in Denmark. The clinic is divided into separate teams, and the ‘depression team’ has an annual intake of 285 patients. Patients offered treatment in the clinic have moderate to severe depression and can have single episode or periodic depression. All patients are referred to the clinic by their general practitioner or a private psychiatrist through the region’s central referral unit. When entering the clinic, patients begin with an individual intake assessment with a psychiatrist or a psychologist before their case is discussed at a treatment conference. Patients meeting the diagnostic criteria for depression are generally offered 14 sessions of group CBT, with eight patients and two therapists in each group.

Patients with depression (single episode or periodic) treated in the outpatient clinic have moderate to severe depression or complicating factors like comorbidity or long term occupational failure. In the years 2019-22 the average WHO-5 score was 20 (N = 473) at onset and 45 at end of treatment, and 25% of the patients drop out of treatment. Unpublished data from a randomized controlled study conducted in Danish mental health outpatient clinics across Denmark in 2016-19 (Reinholt et al., 2021) includes 259 patients with depression as first or secondary diagnosis. In this data set, WHO-5 is highly correlated with BDI-II and a WHO-5 score of 20 points corresponds to a BDI score of 30 while a WHO-5 score of 45 corresponds to a BDI score of 22. Hence, overall the patients improve from severe to moderate depression during treatment. The remission rate (patients with BDI < 19) in the study by Reinholt et al. was 35% across all depression severity levels. The remission rate of the participating patients in the current study was also low: four of the patients had BDI scores corresponding to severe depression at end of group therapy, three patients’ scores corresponded to moderate depression and only five patients’ scores indicated that they had remitted, scoring below BDI 19.

### Recruitment

All patients assigned to three consecutive CBT groups for depression were asked to participate in the intervention study. Twenty-four patients were invited, and nineteen patients agreed to participate. Six of the nineteen patients dropped out of therapy before the last session. Fifteen patients, three of whom had dropped out, agreed to participate in post-intervention interviews. The patients were introduced to the project by a psychiatrist or therapist in the outpatient clinic and gave consent that the first author could contact them and invite them to an inclusion interview. In this interview, the patients would fill out an informed consent form, be introduced to the app for ROM, and the first author would conduct an assessment for research eligibility to confirm depression as the primary diagnosis and identify possible comorbidities. The assessment included the semi-structured diagnostic interview the Mini International Neuropsychiatric Interview (MINI) (Sheehan et al., 1998).

The therapists were selected for the study through purposeful sampling where we wanted therapists represented who had more than five years of experience with psychotherapy and more than three years of experience with group CBT. The selection criteria matched five of the therapists working at the clinic, and they all agreed to participate in the study. One of the therapists participated in two CBT groups and therefore participated in two separate interviews.

### Participants

Our data consist of 21 interviews, including 15 patient interviews and six therapist interviews (see Table 1 for an overview of patient characteristics). The therapists were all women between 33 and 54 years old; three were psychologists, and two were nurses with psychotherapeutic training. They had four to ten years of experience with group CBT. Due to the small number of therapists working at the clinic, we have chosen not to present individual characteristics to ensure their anonymity.

**Table 1:** Patients interviewed

Patients interviewed						
Pseudonym	Sex	Age range	Comorbid diagnoses according to MINI diagnostic interview	Occupation	Progress and adherence	Self-defined problem in PSYCHLOPS**
Lisa	F	20–28	Social anxiety	On sick leave*	OT	“I have no energy”
Silvia	F	40–50	-	Studying	Drop-out	“Feeling restless and doing to many things”
Ruth	F	50–60	-	On sick leave*	OT	“Lack of energy”
Lola	F	18–28	-	Full-time job	Drop-out	“Feeling hopelessness”
Rose	F	50–60	-	On sick leave*	NOT	“Lack of joy”
Robert	M	50–60	-	Job-seeking	OT	“To start the day”
Sophie	F	18–28	-	On sick leave*	NOT	“Not being happy or satisfied at any time”
Nadia	F	40–50	-	Studying	NOT	“I am not happy”
Noah	M	18–28	-	On sick leave*	NOT	“I do not generally feel happy”
Samuel	M	40–50	PTSD	Part-time job	NOT	“Not wanting to do anything”
Kathrine	F	40–50	Panic disorder and agoraphobia	Job-seeking	NOT	“Feeling discomfort”
Anna	F	18–28	Panic disorder and agoraphobia	On sick leave*	OT	“I do not have energy to do the things that used to make me happy”
Julie	F	30–40	-	Unemployed	Drop-out	“Extreme tiredness”
Justin	M	20–30	Social anxiety	Studying	NOT	“Not feeling joy”
Samantha	F	40–50	-	On sick leave*	NOT	“Fatigue”

*More than three months

** The PSYCHLOPS information seems uniformly part of core depressive symptomatology, and cannot help us explain lack of therapy alignment in this particular study

Patients participated in ROM throughout the course of treatment. Four of the fifteen interviewed patients were On Track (OT) during the entire therapy course (see definition of OT and NOT below). We wanted to include both patients who were NOT during the therapy course and patients who were OT during the therapy course, since both types of experience are valuable for identifying patient needs and inform content for individual add-on interventions.

87% of the patients were taking antidepressants and had regular consultations with the senior psychiatrist.

### Researchers

The research group consists of both CBT therapists and psychodynamic therapists, five of which are employed at mental health clinics. Five of the authors are psychologists and one is a psychiatrist. Three of the authors are professors and one is a PhD student. JRG took the lead on gathering data and was supervised by SP, CM and SMA. EBB and KM work as clinicians in the clinic where the study took place. Based on clinical and research experience, all authors expected that the informants would express that the therapy could be more adjusted to the patients’ individual needs.

### Intervention

Following regional guidelines, the psychotherapy offered for depression in the outpatient clinic is standardized group CBT. The therapy consists of 14 two-hour weekly group sessions and three individual sessions distributed before, midway through, and after the therapy course. The treatment follows a protocol inspired by the work of Melanie Fennell (Hawton et al., 1989) and is adapted to group therapy (Due Madsen, 2008). The manual is structured according to the plan presented in Table 2. As evident from the group session schedule, this intervention has a solid focus on psychoeducation and learning about depressive thought dynamics, as well as an action-oriented here-and-now experiment approach. From session nine the focus shifts to more of a cognitive restructuring approach, including identification of underlying assumptions.

**Table 2:** Overview of group sessions

Overview of group sessions	
**Session 1**	Introduction to group therapy, information on depression, and presentation of activity tracking and evening therapy
**Session 2**	Activity planning and nourishing activities
**Session 3**	The vicious depression cycle and distraction techniques
**Session 4**	A 4-column schedule including situation, negative automatic beliefs, emotions/ body, and behavior
**Session 5**	Cognitive distortions
**Session 6**	Alternative thinking and challenging negative automatic thoughts
**Session 7**	Behavioral experiment
**Session 8**	Recap of alternative thinking
**Session 9**	Uncovering unhelpful rules for living
**Session 10**	Changing unhelpful rules for living
**Session 11**	Behavioral experiment with new rules for living
**Session 12**	Continued behavioral experiments with new rules for living
**Session 13**	Relapse prevention plan
**Session 14**	Ending group therapy

### Measures

Patients participated in three consecutive CBT groups for depression with ROM and feedback (see ROM questionnaires in Table 3). The questionnaires were included as part of the study and are not used as a standard practice at the clinic. The patients replied to short questionnaires on a smartphone app (Monsenso ©) shortly before and during the group CBT course. If a patient was NOT, the therapists were informed and encouraged to intervene with assistance from a slightly modified version of a Clinical Support Tool (CST) applied in a study of ROM and CST (Koementas-de Vos et al., 2018). The therapists did not have previous experience with ROM and were trained in the interpretation of the patients’ feedback and the use of the CST at two two-hour workshops.

**Table 3:** ROM Questionnaires

ROM Questionnaires	
Overall Depression Severity and Impairment Scale (ODSIS)	Five items that aim to measure the severity of and impairment due to depression. The scale has demonstrated discriminant and concurrent validity. It addresses depression frequency and severity, level of engagement, work/school/home interference, and social interference on a five-point Likert scale (Bentley et al., 2014). The scale was reversed to make the graphic illustration of the progress trajectories similar to the other questionnaires.
Significant life events in the past week	Six items that aim to clarify if the patient has experienced any significant events considering finances, job, family, housing, intimate relations, or overall life situation during the past week. The questionnaire was designed for a randomized controlled trial concerning group CBT (Reinholt et al., 2021).
Two items from PSYCHLOPS	Two questions that are rated on a 6-item scale. The items seek to include patients’ perspectives on psychological distress and let them define the problem they want to improve (Turvey & Fortney, 2017).
Homework Tracker	Consists of one item concerning patients’ adherence to homework during the preceding week. It was designed for a previous study (Reinholt et al., 2021).
Group Questionnaire – Brief 12-item version	Aims to measure three relational domains in group therapy: positive bonding, positive working, and negative relationships (Krogel et al., 2013). The original questionnaire is a 30-item, 0–7 Likert scale that has demonstrated good construct validity. In the present project, we used a 12-item version (Tsulukidze et al., 2015).

The questionnaires appeared on the patients’ smartphone apps with a notification the morning before therapy, except the ‘group questionnaire’ which appeared just after therapy ended. Every questionnaire had a text field where the patients could add comments. Patients were considered NOT if they scored 3 ODSIS points lower than baseline at any point in their treatment or if no change (i.e., < 2 ODSIS points) occurred for three sessions in a row. The first author would give the therapists a NOT-report if a patient was considered NOT based on these criteria. The NOT-report would include negative responses to ODSIS, the group questionnaire, PSYCHLOPS items, or homework tracker. If the patient had inserted comments, these would also appear in the report. The therapists were also provided with access to the Monsenso© web portal, where they could follow all patients’ progress in a graphic display. A more thorough presentation of the study design can be found in the published research protocol for the current study (Gryesten et al., 2022).

### Interviews

After the therapy course had ended, participants were invited to individual interviews, which lasted 50–90 min. Sixteen interviews took place in an office in the outpatient clinic, and five patient interviews were by phone. The interviews were conducted by the first author and transcribed verbatim. Interviews were semi-structured and were based on separate interview schedules for patients and therapists (see interview schedules in *supplementary material)*. The interview schedules were developed by four of the authors and tested in a pilot interview. Afterwards, they were revised based on the interviewer’s experiences with the interview schedule. As an example, we included more interview questions oriented toward lived experiences such as “Can you give an example from your daily life of how the treatment has changed you?” This flexible approach enabled greater interview depth by optimizing the interviewer’s capability to encourage the participants to elaborate on their subjective experiences. The interviews began with explorative and open-ended questions and became more focused on specific themes as the interview progressed. Patients were asked about their experiences during their own therapy course and therapists were interviewed about their experiences of specific patients’ therapy courses. After asking broadly how the participants experienced therapy and about their own/a specific patient’s progress, the interviewer provided visual graphs from the participant’s ROM. First, we asked the participants how they understood the progress trajectories, and afterwards we asked about specific points on the graphs where the patient was OT or NOT, in order to examine what was helpful and unhelpful at these times and what patients needed when they were NOT. The patients’ comments in the ROM text fields were presented to support their retrospective memory of their thoughts and feelings during therapy. The interview schedules do not solely include questions concerning personalization, but cover the patients’ experiences of the therapy and ROM more broadly.

Before conducting the study, we described three possible add-on interventions based on clinical experience and former research studies: “Unrecognized psychopathology of the patients – managed by therapeutic assessment and, potentially, supplementary pharmacological treatment; Problems in the patient’s environment and social life outside therapy – managed by network-based problem-solving; Therapy hindering psychological processes – managed by collaborative case-conceptualization” (Gryesten et al., 2022, p. 4). The suggestions for add-on interventions were presented to the participants at the end of the interview to get their perspectives on their relevance. In the analysis, we based our findings on the exploratory part of the interviews, where the participants put forward their personal experiences and preferences before the interviewer mentioned the three previously proposed add-on interventions.

### Analytical Approach

The study’s purpose calls for an exploratory qualitative design in which participants’ experiences are examined. A qualitative approach is particularly apt in studies that aim to explore new areas or discover novel knowledge (Brinkmann & Kvale, 2008; Willig, 2013). We systematically applied a hermeneutic-phenomenological thematic analysis. The phenomenological approach refers to our preparation and ambition to allow people to relate first-person lived experiences in the interviews (McLeod, 2011). However, we recognize that we, as researchers, are situated in a particular context and that the research project is pragmatic in that it is directed at contributing to perceived knowledge gaps in a specific clinical field. This preunderstanding (Gadamer, 2013) and situatedness will be part of the interpretation of data, both in interviews and in data analysis, giving the study a hermeneutic epistemological stance. For specific systematic analytic procedures, we were inspired by Thematic Analysis (TA) (Braun & Clarke, 2006), which fits our methodological stance.

We implemented two analytic processes in relation to the data. The first focuses on perspectives on add-on and personalization interventions and is the scope of the current paper. The second relates to general group psychotherapy experiences and will be published elsewhere. As mentioned, the current study focuses on what informants miss in group CBT. Therefore, we have omitted other findings from the interview data such as what it was like to look at their change trajectory graphs and how the informants experienced using ROM. These experiences will be included in a future mixed-methods multiple case study. Therapist and patient interviews were analyzed in a joint process since they provide different perspectives on patient needs. Patients offered their subjective experiences and understanding of their own progress and needs, while therapists offered an observer perspective on specific patients’ progress and needs. When identifying patient needs, we believe that patients’ experiences are indispensable, but the therapists’ clinical perspective is important for understanding the limitations of a standardized framework. Throughout the analysis, our aim was to highlight both the similarities and divergences in perspective between therapists and patients.

The analysis was inspired by the six-phase approach proposed by Braun and Clark (2006), but adapted by including the entire research group to enhance credibility. The transcribed interviews were entered into NVivo. The process followed the following steps:

1. The first author read through the transcriptions and listened to the audio recordings, thereby familiarizing herself with the data and noting ideas.
2. Initial codes were added systematically and often line by line. These were descriptive and were named, e.g., “wants more contact with therapists” or “felt anxious in the group”.
3. Codes were grouped by descriptive similarity, and developed into tentative themes. For example, the codes “difficult relationship with husband” and “lack of understanding from family” were sorted to be part of the preliminary theme “Involvement of relatives”.
4. In order to strengthen confirmability, a research assistant listened to the interviews, read the transcripts, and noted if she disagreed with the constructed codes and themes. In each case, the first author decided what to do in relation to suggested corrections.
5. The first author checked that the themes matched the initial quotes.
6. Selected quotes and their preliminary descriptive interpretations were given to a research group consisting of: two professors in psychology, one professor in psychiatry, one psychologist/senior researcher, two clinical psychologists, one academic employee with a Master of Arts, and one psychologist/PhD student. All participants read through the material before a 3-hour meeting, where the themes were discussed and clustered into main themes.
7. During the meeting, the research group also discussed which individual add-on interventions the findings might suggest to be relevant for NOT patients. Afterward, the iterative process of qualitative analysis continued, and the first author adjusted the themes with oversight from three of the co-authors.
8. The report was written and relevant quotes were selected and translated from Danish to English.

### Findings

We identified twelve sub-themes that were clustered into five main themes (see Table 4).

**Table 4:** Main themes and sub-themes

Main themes and sub-themes		
Main themes	Sub-themes	Found in interviews with*
1. Individual attention	• What is at stake	15 patients and five therapists
	• Talking about sensitive subjects	Six patients and three therapists
3. Psychological exploration	• Understand me better	12 patients and all therapists
	• Life story	Five patients and two therapists
5. A focus on the patient’s life outside of therapy	• Involvement of relatives	12 patients and three therapists
	• Current strains and crises	Five patients and four therapists
	• Social services & institutional support	15 patients and six therapists
8. Extended assessment	• Assessment	Two patients and four therapists
	• Comorbidity	Nine patients and five therapists
	• Cognitive challenges	One patient and four therapists
11. Agreement on therapeutic tasks	• Motivation	12 patients and four therapists
	• Homework	Three patients and three therapists

* Out of a total of 15 patient interviews and 6 therapist interviews

### Description of main themes

In the following, we present the five main themes and associated sub-themes. The patients’ and therapists’ statements will be integrated.

### 1. Individual Attention

A significant finding emerged regarding the patients’ perceptions of recognition and individual attention from their therapists. Patients reported that they highly valued instances in which their therapists talked with them outside of the group therapy setting. Additionally, therapists reflected on the potential therapeutic benefits of having individual supplementary sessions when needed, while acknowledging that this was not consistently implemented in their clinical practice. Furthermore, they noted that, in retrospect, they believed that such individualized attention would have been beneficial for certain patients.

### What Is at Stake

In the sub-theme ‘What is at stake,’ it appears that some patients needed a discussion where the therapist would clarify if the patient needed something. Tobias, who was persistently NOT, described this in the following way: “like a status check, how are you and just like, why are you maybe not getting so much out of this, or are you about to drop out and what can we do to change it” (Tobias). Based on the ROM and his own awareness of his mental state, Tobias was not in doubt that the therapists knew he was not getting better during the group course. However, he was frustrated that the therapists did not verbalize this and explore how it could be changed.

Some patients reached out themselves for additional contact with the therapists, while other patients were not capable of doing this, which could lead to self-blame for being passive: “they [the therapists] would not know if I didn’t tell them, and I didn’t” (Rose). Some therapists stated that they informed patients that they were available if anybody had concerns or information they did not feel comfortable sharing in the group. However, this solution appeared to be inadequate for patients who find it hard to reach out. Instead, these patients would need the therapist to check with them directly how they experienced therapy. Some therapists also acknowledged that it might have been helpful if they had reached out to patients when they were NOT. For example, a therapist described a therapy course with a patient who, in the ROM, had rated the therapeutic alliance as low:

With him, I paid extra attention the next time he came. That he had answered like that, and maybe I was curious – how do I express myself? If that is what had offended him or something. But in the end I should maybe have taken him aside and said – I see that you have answered like this. What is it about? (therapist, discussing NOT patient).

The therapist was aware that the patient was dissatisfied with the therapeutic alliance. However, she only used this information indirectly, instead of as a stepping-stone to explore how the patient was experiencing therapy and perhaps repair the alliance.

Therapists also mentioned specific cases where they had initiated individual contact with patients. For example, a therapist mentioned that she had given an extra individual session to an introvert patient in the group: “She was that type where you need to talk with her one-to-one in order to know – how are you really?” (therapist, discussing NOT patient). This underlines that in some cases there is a need for individual sessions in combination with group therapy, since it can be challenging to achieve sufficient contact with the patient in a group setting.

### Talking About Sensitive Subjects

Some patients felt that there were specific issues they were more comfortable discussing in private sessions with a therapist rather than in the group setting. For example, one patient stated that: “You can say other things [in individual sessions] than you can in a group session. More personal things about kids and my husband” (Nadia). It seemed the patients experienced the group as a public room, in contrast to individual therapy which was seen as a more private space. This also appeared in the language used, where some patients would refer to the group therapists as “teachers” (Ruth, Sophie, Samantha) and to the group therapy as a “class” (Robert, Ruth, Carla). In contrast, all patients would mention the therapists by name when they referred to the individual sessions they had with the same therapists.

The therapists often spoke positively about patients who shared personal stories in the group. For example, one therapist said about a patient: “She is cool because she talks very openly about what bothers her and what challenges she has*”* (therapist, discussing NOT patient). These patients had the advantage that they shared their thoughts and feelings, and it seemed easier for the therapists to understand them and their needs. Accordingly, when the other therapist from the same group talked about the same patient, she reflected: “It might have been good to have one or two individual sessions when her vulnerability was very visible, in order for us to talk with her about it first and then continue working with it in the group*”* (therapist, discussing NOT patient). The quotes demonstrate that, on the one hand, therapists found it beneficial when patients discussed sensitive topics in a group setting, as it allowed them to better understand the patients. On the other hand, in the given case, the therapist seemed to believe that group CBT alone was not sufficient to provide the necessary support for the patient, but could be advantageous as a follow-up to individual sessions where psychological interventions were initiated.

### 2. Psychological Exploration

In this main theme, it appeared that both patients and therapists found it important that the therapists had a thorough understanding of each individual in the group. Patients and therapists highlighted the value of exploring individual patients’ experiences and psychological mechanisms. However, it seems that the current format might not be optimal for exploring unique individual experiences thoroughly. This can have consequences for the relational bond between patients and therapists, since some patients expressed that they were not getting the understanding and exploration they needed.

### Understand me Better

Some NOT patients did not experience that the therapists sufficiently understood them as individual and separate persons, but rather that they were more seen in the light of the depressive symptoms they had in common with the other patients. All patients mentioned that the most helpful part of the therapy was to be mirrored by other group members and meet others in similar positions. However, at the same time, some of the patients mentioned that they wanted to be better understood by the therapists, indicating this with statements like: “Well, they might have an understanding of all that about anxiety and depression, but maybe not really of me as a person” (Tobias). Some therapists also indicated difficulty understanding specific patients: “It was very difficult for me to know how he felt. Because he was so guarded against something and evasive and… not a part of the group” (therapist, discussing NOT patient). The therapist states that the patient was not part of the group even though he was physically present. This might reflect the therapist’s limited capability for responsiveness to the patient. All therapists mentioned that to produce psychological changes in group CBT, they needed to have a thorough understanding of the individual patient:

It means a lot that we have acquainted ourselves with the patients’ individual histories so that we not only conduct group therapy but also understand specific individuals in the group. It is significant to intervene when there is avoidance. And try to make the patient hold on [even though it can be painful]. (therapist)

The therapist then underlined the importance of a well-conducted case conceptualization and that this preunderstanding enables the therapist to adjust the therapy individually in a group setting. However, the patients’ experiences illustrate that this intention may not always have been successfully transformed into action. In addition, some of the therapists’ experiences of specific therapy courses highlight that the therapists’ understandings of patients are sometimes inadequate. Within this sub-theme, there are divergent experiences in relation to whether the therapists can achieve a comprehensive understanding of the individual patients within standardized group CBT.

### Life Story

It appeared that some patients wanted to talk about their past and how past events affected them in the present. For instance, a young man specified that he needed: “Something about childhood traumas. Because I have talked with my older brother a lot about that [and] we both feel there was some neglect” (Noah). Some older patients expressed this need as well, e.g., a patient who was now in her second course of group CBT and not experiencing any change. When asked what she needed, she replied:

I can feel that there is something that takes much space in my head that I cannot control, there are many things from my past or my previous life that are pressing, and I would have liked if there were some more room for that. I am aware that cognitive therapy is about the present as I understand it. But I feel that I would also have liked to be able to go back in time. (Rose)

Thus, this patient (along with several others) seems to understand the concept and scope of the treatment she has participated in, yet clearly expresses her specific need to talk about her past and finds it problematic that there is not room for doing this within the treatment format.

### 3. A Focus On The Patient’s life Outside Of Therapy

This main theme reflects that patients and therapists found it helpful to involve people or perspectives from the patients’ networks. Involving relatives, addressing current strains and crises, and creating bridges between therapy and other arenas in a patient’s life can have a positive impact. These findings support a holistic approach to mental health treatment, which recognizes that mental health not only involves the individual, but should consider the patient’s whole life situation to create a comprehensive plan for improvement. The theme is not only relevant in group CBT since the impact of environmental stress on treatment engagement appears in psychotherapy in general. However, in group CBT the patients might not always have the chance to discuss their current challenges if they are not relevant for the focus of the CBT manual.

### Involvement of Relatives

The sub-theme ‘Involvement of relatives’ concerns the need expressed by many patients to involve relatives in the therapy. One of them was a young woman who wished to involve her parents:

There was much pressure on me because they [her parents] had so many questions about how they should take care of me (…) So I think it was challenging to have two parents looking at me with searching eyes, wanting me to open up and answer all of the questions they had (…) And I just think there are some situations I could have avoided with my parents if they had gotten some more information. (Sophie)

Therapists also mentioned the importance of including the patients’ relatives, either by talking about them or by including them in person in one or more sessions. For example, a therapist stated: “I noticed that when she had relational problems, it added to her depressive symptoms” (therapist, discussing NOT patient). This recognition that patients cannot be understood in isolation from their relationships informs us of the importance of discussing their social life and, on occasion, involving their relatives in the therapeutic process. A difference between therapists’ and patients’ preferences in relation to involving relatives was that therapists typically mentioned the potential benefits of informing relatives about the patient’s treatment and educating them about depression, whereas the patients would request a more comprehensive therapeutic effort that dealt with issues in relationships.

### Current Strains and Crises

Some patients described events outside of therapy directly leading to deterioration. For example, when looking at the curves in the ROM of depression symptoms during group therapy, a patient stated:

There are tangible reasons why it goes up and down in the course of therapy. External things knock me out because I am not so strong. Instead of it just being my depression, I think it is things happening in my life. Negative things that leave me feeling defeated. And then combined with my depression then [makes falling whistling sound]. (Samantha)

The quote demonstrates that the patient is affected by events happening in her life, and she indicates that her lack of resilience causes current events to have a detrimental effect on her. This belief underlines that it would be relevant to address the events in her life directly in therapy in order to create change in depressive symptoms.

### Social Services & Institutional Support

Some of the patients described how institutional demands would put pressure on them and make it difficult to focus on therapy:

I have been going to school while being in therapy, which has been very difficult for me because I have been under much pressure in school, and those things I was supposed to do, I have not been able to do because of my depression. And then it has been difficult for me to work with myself [in therapy] because there was a lot of pressure there [in school]. (Sophie)

The statement demonstrates that expectations from school and therapy can collide when there is no connection between the two arenas. Therapists would also express the need for better collaboration with the municipality, workplace or school. One of the therapists reflected on the therapy course of a NOT patient who was still severely depressed after therapy and was struggling with loneliness: “I think what would have made sense was if we had pushed more to get the local authority up and running. We should probably have spent individual sessions on that, through network inclusion or something like that” (therapist, discussing NOT patient). In this case, the therapist thought it would have been more helpful to focus on social support than therapeutic strategies.

### 4. Extended Assessment

This main theme demonstrates the need for more in-depth assessments, considering comorbid conditions, and addressing cognitive challenges as part of the therapy process. The group format seems to have some limitations when comorbidity is severe, and it is experienced as a limitation that all of the patient’s symptoms and issues cannot be addressed in the group. The narrow focus on the symptoms of depression is especially experienced as challenging for the therapists in more complex cases.

### Assessment

Some patients expressed a need for more assessment time during the therapy as they realized that the category “depression” did not fully cover their struggles. For example, one patient mentioned that a diagnostic assessment during the therapy course might have been beneficial, since the depressive symptoms were related to her blaming herself for her anxiety symptoms:

To me, it would be good to find out why I have reacted to, for example, social anxiety, because for almost 29 years I have been knocking myself in my head and telling myself to pull myself together. So maybe it could be easier for me to accept why I react as I do [if I got another diagnosis]. (Lisa)

The therapists also mentioned cases where they thought an assessment would have been helpful:

She is one I have been thinking about afterward. Because she is actually one we could have assessed for an avoidant personality disorder. She is such a type that I have been in doubt if it was the right thing that I did not look more into it? (therapist, discussing NOT patient)

It seems that the therapist was aware during therapy that the patient might have been placed in the wrong treatment. However, she did not take action on this thought, and whether the patient would indeed have benefitted from a thorough assessment is unknown. This underlines the potential benefit of having the option of an extended diagnostic assessment as one of several alternative interventions when patients are NOT. Without such guidance, therapists might not act even though their clinical experience tells them to.

Therapists primarily mentioned patients they suspected met the criteria for personality disorders, whereas patients mentioned that they suspected they might be affected by stress, anxiety, and (in a single case) autism. Therapists also mentioned that clinical assessments could give room for more person-centered psychoeducation.

### Comorbidity

Patients would sometimes suffer from symptoms not pertaining to depression, which could affect group therapy. One patient mentioned that her anxiety inhibited her capability to engage in the group and impacted on her outcome:

I just knew that if I said one word, I would collapse totally. So I was sitting and doing breathing exercises and drank water and tried to think of something else and (exhales) (…) I didn’t say anything. Because when you are sitting with so many people, then I should have said ‘can we please take a break together outside?’. But then it would all have been too much for me. So I tried to kind of hide it in order to be able to stay there because otherwise, I knew I would not be able to stay in there. (Carla)

The patient deemed her emotions unsuitable for the setting. The CBT protocol primarily focuses on depression-related behavioral activation and cognitive re-structuring, leaving other problems untouched. The patient might experience that her feelings belonged to another type of treatment. Some therapists acknowledged the specific treatment focus. Other therapists expressed that it was difficult to contain patients’ challenges related to comorbidity in the group and questioned the narrow symptom focus:

In his case, the frustration is the format of the treatment – that this is depression treatment. And that we almost have to, to put it a bit bluntly, close our eyes to his PTSD. That we cannot intervene on the symptoms in that setting. Even though we know it’s a part of him. […] And we can see, for example, in the behavior-focused part of treatment that it is not possible in reality, and that can be frustrating. (therapist, discussing NOT patient).

The quote indicates that the therapist needs room to personalize the treatment for the individual rather than the specific diagnosis, but finds it impossible within the current treatment program.

### Cognitive Challenges

Some therapists mentioned a need to examine the degree of cognitive challenges, such as memory and comprehension difficulties, in specific patients and follow up with an intervention. For example, one therapist mentioned that in some cases, she was unsure how the patients’ cognitive challenges impacted the treatment, which affected her confidence in how to approach the patient: “I think it can be difficult when they have cognitive challenges. How much can we challenge them? How much pressure can we put on them? And especially when it’s also a depressive symptom” (therapist). The therapist appears indecisive with regard to addressing cognitive challenges and whether to assess the extent of cognitive deficits. Notably, the patients did not express any inclination to prioritize cognitive challenges. Rather, they expressed an accepting attitude towards them.

### 5. Agreement on Therapeutic Tasks

This main theme reflects the need for negotiation between therapist and patient concerning the therapeutic approach. Due to the pre-determined framework of manual-based group CBT, therapists would mention the importance of being able to do CBT-specific tasks, especially homework. However, some patients expressed that they would prefer a more unstructured format. According to the therapists, the performance of homework is a significant aspect of CBT. However, patients’ perceptions of the relevance and usefulness of the homework vary.

### Motivation

When therapists explained why specific patients profited from therapy, they would always mention motivation. For example, one therapist spoke warmly about a male patient who felt a lot better after therapy: “He was highly motivated. And he was so ready for a new change. (…) And he is a man of action, so CBT is really something for him. So very suitable for therapy and very ready. Very engaged” (therapist, discussing OT patient). In contrast, therapists often mentioned a lack of motivation when the interviewer asked why specific patients did not benefit from therapy. Therapists also mentioned that lack of motivation could have the consequence that the patient would not be offered as many therapy sessions:

I would actually also have been fine with giving that to him [an individual session]. But I don’t know why I didn’t do that. I would actually have liked to do that. (…) I think I have been in doubt from the beginning if he was motivated. (therapist, discussing NOT patient)

The quote indicates that patients capable of demonstrating motivation might have a better chance of receiving more intensive care than others in the group. Showing commitment to therapy, therefore, is not only crucial for the chance of profiting from the therapeutic tasks, but also important for motivating the therapists to give one the best treatment.

Two patients mentioned explicitly that they felt the therapists did not think they showed enough motivation in therapy. One of these patients stated: “I sometimes had the feeling that the therapists were – not mad at me, but that they did not think I did well enough with participating and doing that homework” (Tobias). The other patient mentioned that when she had the feeling that the therapists thought she was not performing well enough or showing enough motivation, it would be helpful if the therapists would talk with her about her individual experiences in order to get a better understanding of her actions:

I missed more room to talk freely. It was all about following the book. It can also be difficult for them to understand, why have you not been doing your homework? Is it just because you don’t prioritize it? No, it’s not necessarily because I don’t prioritize it. (Samantha)

The quote indicates a disagreement about the therapeutic tasks and structure of therapy. The therapists followed the manual whereas the patient prefers a more unstructured approach. This disagreement can potentially be misinterpreted as lack of motivation.

### Homework

In line with the above, in the sub-theme ‘Homework’ it appeared that the therapists would emphasize the patients’ capability to do the homework. For example, when asked how she experienced a therapy group, one therapist replied that it was a difficult group because:

Many of them did not do the homework, and for a lot of them they wouldn’t attend one session and then they came to the next session and stuff like that. So there has been unstable attendance and unstable homework. (therapist)

In the same way, therapists would emphasize successful therapy courses where the patients were good at doing the homework. Some patients mentioned that they would not always find the homework relevant to their issues, and it felt pointless to do the homework then. Other patients explained how they adjusted the homework so that it would fit them better. Further, some patients mentioned that the order of the homework exercises did not fit their personal needs:

The homework is approached from page one to page… well, so it’s totally chronological. But we are not chronological as humans. So in that way, there could have been some homework later in the course that would have benefitted me earlier and vice versa. (Samantha)

The patient’s reflection on how the homework progressed indicates that she would have preferred a more personalized way of using the homework. The same patient mentioned that she would sometimes feel that the therapists thought the homework was more important than the individual:

Sometimes we would not have time for that round – what has happened in our lives during the last week? Alternatively, if we did, then it was very minded towards the book. And then it did not make sense to talk about me and my goal. Because in reality it was all about the specific homework. More than it was about me personally. (Samantha)

Thus, it seems the therapists and patient did not agree on the importance of homework tasks. This issue can also appear in individual CBT. However, it seems that in group CBT, the therapists need to focus on many patients and therefore the capability of personalizing the homework might be inhibited.

## Discussion

Summarizing the themes and descriptive content, our findings suggest that personalizing group CBT might be valuable since patients may experience that important needs are not being met in standardized treatment. The analysis delineated five main themes: (1) Individual attention, (2) Psychological exploration, (3) A focus on the patient’s life outside of therapy, (4) Extended assessment, and (5) Agreement on therapeutic tasks. The findings open up many potential considerations on how to accommodate patients’ individual needs in manualized group CBT. The following section presents a selected discussion of some of the aspects of the findings.

Our results overlap with previous findings on helpful and hindering aspects of psychotherapy. For example, in their meta-analysis of helpful and hindering aspects in individual psychotherapy, Ladmanová et al. (2016) identify the theme, “Feeling Heard, Understood and Accepted”. In the current study, some of the patients expressed a need to feel understood, which is described under the sub-theme “Understand me better”. Also, the hindering factor “Having Difficulties Disclosing”, which appeared in the study by Ladmanová et al. (2016), has some overlap with our main theme “Individual attention”, where the therapists spoke positively about patients who shared personal stories in the group. The helpful and hindering aspects identified in the meta-analysis are reflected in the unmet needs identified in the current study. However, a difference is that the current study concerns manual-based group CBT, and it appears that many of the needs of patients could potentially be met with individual add-on interventions, to make room for helpful events.

In the main theme ‘Individual attention’, it appeared that both patients and therapists experience that it is helpful if therapists take an individual’s ‘status’ and check whether the patient feels on the right track. The combination of group and individual psychotherapy is standard practice in other treatment programs, such as Dialectical Behavior Therapy (Linehan et al., 1991), which is typically directed at other populations than depression. Also, a qualitative study concerning group CBT for eating disorders found it important to combine group and individual sessions (Laberg et al., 2001). The results indicate that group psychotherapy potentially faces an inherent challenge in not meeting patients’ need to be treated in a personalized manner on an individual basis. Compared to individual psychotherapy, it is a notable advantage of group psychotherapy that it offers, e.g., interpersonal support, normalization, and group cohesion (Burlingame et al., 2001; Kivlighan & Kivlighan Jr, 2016). Nevertheless, a challenge in group psychotherapy is that it is more difficult to be understood as an individual in a group setting. This is particularly evident in standardized group therapies, which follow a “one size fits all” approach. In understanding the need for individual attention within a group psychotherapy context, we can apply the theoretical concepts of need for agency and need for relatedness (Bakan, 1966; Newhill et al., 2003). Relatedness refers to being part of a community or union, whereas agency refers to being distinct from it and more autonomous. There is a dialectical relation between the two concepts, and in all relationships, especially in therapy, we will search for relatedness while striving for agency. The results of this study should therefore not be understood as an indication that patients and therapists, by definition, prefer individual therapy rather than group therapy. Instead, we will argue that the results call attention to the specific and distinct needs that patients might have during group therapy while at the same time profiting from the group-specific factors.

In the main theme ‘Psychological exploration’, some patients conveyed that the therapists did not sufficiently understand them. Concurrently, some therapists mentioned cases where they had not achieved a complete understanding of the patients. This might indicate that taking time to uncover the individual patient’s challenges and strengths, in collaboration with the patient, might be helpful for some patients during group CBT. In line with qualitative research on individual CBT for depression (Barnes et al., 2013), some patients needed a focus on their past and wanted to be able to talk about past traumas that affected them in the present. This need for psychological exploration could, to a certain extent, be met by focusing on a thorough case conceptualization. Case conceptualization is considered a core element across most therapeutic approaches (Eells, 2022), and its purpose in the current context is to provide information that can aid the therapist and patient in the group therapy setting by supporting helpful processes such as recognition and self-understanding. A potential issue of focus is whether the standardized CBT group can accommodate the psychological insight that patients might acquire through the case conceptualization, or whether the insight will be left without further therapeutic intervention. For example, if the therapist and patient recognize the impact of childhood neglect, there might be limited options as to how much emphasis the problem can receive in group CBT.

The main theme ‘A focus on the patient’s life outside of therapy’ reflects that patients and therapists noted the importance of involving the patients’ network. Patients and therapists experienced that external events in the patient’s life could influence therapy, and patients experienced that they sometimes missed a stronger connection between their psychiatric treatment and the other public arenas they were part of (e.g., family, school, and job agency). The results are comparable with previous research, which has found that psychosocial factors – such as ongoing severe stress or relational problems – may impair CBT outcomes (Moorey, 1996). Evidently, individuals in psychotherapy cannot be understood independently from their context and relationships (Wampold, 2013). By employing a context-oriented add-on to group CBT, it would be possible to initiate social interventions that might strengthen the patient’s capability to focus on the treatment. Previous research found an effect of systemic involvement of relatives in psychotherapy for patients with depression (Asen & Jones, 2018). However, it is probably important to ensure that the add-on is not a separate intervention with no connection to the group therapy. To meet the need for a focus on the patient’s external life, initiating change in relational dynamics or creating more collaboration between public arenas might help the patients be more able to focus on the group therapy.

The main theme ‘Extended assessment’ reflects that therapists often suspected that the patient might meet the criteria for another diagnosis when patients were NOT. Some patients also expressed the need for an assessment or wonder whether treatment for depression was the most suitable option. Comorbidity was sometimes experienced as obstructing therapy, and the therapists stated that it was difficult to psychoeducate on issues related to comorbid challenges within the framework of a manual-based group treatment. Therapists mentioned that they sometimes suspected that cognitive challenges impacted the patients’ capabilities to participate in therapy and do the assigned homework. The results are in line with previous findings on variables that may impair outcomes in CBT, including comorbidity (Whitfield, 2010). An add-on intervention inspired by Therapeutic Assessment (Finn, 2011) might lead to the therapist and patient obtaining a better insight into the patient’s psychopathology and being able to include this information in the group therapy, or suggest another treatment program if relevant. Therapeutic Assessment has previously been suggested as relevant for NOT patients and is distinguished from a traditional diagnostic assessment since it is more patient-directed and includes an element of therapeutic intervention (Fantini et al., 2022). A diagnostic assessment can potentially lead to a new diagnosis that indicates that group CBT for depression is not a suitable treatment. In these cases, the persistently NOT patient should be offered another type of treatment. However, in other cases, while the assessment might reveal other symptoms unrelated to depression, the patient might still be able to profit from group CBT for depression. In these cases, the new information and subsequent psychoeducation is likely to improve the treatment of the patient’s depressive symptoms and mean that the patient will be more able to apply the therapeutic tools in group CBT for depression.

Finally, the main theme ‘Agreement on therapeutic tasks’ demonstrates the value that therapists place on the patients they define as motivated and the challenges that can arise when working with patients that the therapists see as lacking motivation. When patients were not doing homework, this was stressed by therapists as an obstacle to therapy, while some patients mentioned that they thought homework took too much focus in therapy. In some instances, not doing homework might indicate lack of motivation. However, the patients’ experience that homework took too much focus in therapy demonstrates that patients not doing homework can also indicate a lack of agreement about therapeutic tasks, rather than a lack of motivation for therapy. The results are in line with previous research that suggests that low levels of motivation, difficulty in understanding the CBT model or preference for another model, and lack of capability or willingness to do homework are associated with impaired outcomes in CBT (Barnes et al., 2013; Cramer et al., 2011; Ghaderi et al., 2022; Nilsson et al., 2007). A therapeutic alliance combines a relational bond, agreement on the goals of therapy, and agreement on therapeutic tasks (Bordin, 1983). Thus, the lack of agreement on therapeutic tasks has consequences for the therapeutic alliance, associated with poor therapy outcomes (Arnow et al., 2013). Due to limited options in the Danish outpatient psychiatric services, patients with depression are usually only offered group CBT. Accordingly, the patients and therapists may have to figure out how they can agree on the therapeutic tasks within the CBT format or how the content of the group psychotherapy can be meaningful for the patient, e.g., by adjusting the homework. Personalization of the CBT strategies presented in group psychotherapy might be more feasible within individual add-on sessions than within the group. However, the aspect of agreement on therapeutic tasks in CBT is different from that presented in other therapy formats directions, such as pluralistic therapy (McLeod, 2017), where the treatment to a much larger degree follows the patients’ needs through shared decision-making. Nilsson et al. (2007) conducted a qualitative study comparing CBT and psychodynamic therapy and found that patients who were offered therapy that did not match their preferences were dissatisfied. Implementing an add-on for a patient who prefers another type of therapy might not be a sufficient solution, though it can be viewed as a step in the right direction.

A more general consideration based on the findings of the study is the relative lack of success of the feedback to the therapists. In the current study, the therapists were given information about each patient from the ROM and were provided pieces of advice in the CST text. However, the results demonstrate that even though therapists received this feedback, patients still had unmet needs, as demonstrated clearly in the themes “understand me better” and “agreement on therapeutic tasks”. The results indicate that negative feedback from the ROM about a specific patient should be followed up by systematic attention to the patient in question. Furthermore, it appears to be crucial that the therapists have realistic options to adjust the therapy based on the patients’ feedback.

The study’s findings have important ethical implications. As presented, some patients felt that the therapy they were receiving was not helpful to them and not adapted to their needs. Since the Danish outpatient Mental Health Services are not usually offering different types of therapy, the patients only have the options to continue treatment even though they find it unhelpful or to drop out. As previously mentioned (in the ‘Setting’ section), the alternative of 5–7 individual sessions compared to 14 group sessions might also cause some patients who would prefer individual therapy to refrain from choosing this option since the therapy course is significantly shorter. The clinic’s drop out rate of 25% further indicates that some patients are not receiving the help they need and that more flexibility and alternative therapy options are called for.

## Implications for Personalizing Group CBT

This study has the following implications for the personalization of group CBT for NOT patients:

- The format of group CBT has an inherent limitation that can potentially threaten the relational bond between therapists and patients. Patients may feel that they are not getting the attention they need within the group context, and therapists may feel unable to provide the necessary care and insight within the structured format.
- In the structured format of standardized group CBT, there is a lack of space to negotiate content and approach for the individual patient, which can mistakenly lead to an understanding of patients as not motivated for therapy.
- The diagnosis-specific focus may be a hindrance for the provision of a comprehensive treatment of the patient, and the treatment can accordingly be perceived as inadequate by both patients and therapists.
- Group CBT should be able to address the individual patient’s life situation and allow the therapists to understand the individual in their life context outside of the treatment setting.

Based on these implications, we suggest an operationalization of personalized group CBT. Personalization within group CBT means that each patient is understood as a unique person who has distinct individual needs that can only be clarified through an in-depth dialogue between patient and therapist. The dialogue should take place continuously during the therapy course and encompass the following:

1. Examination of the patient’s needs, preferences, and goals.
2. Regularly assessing the effectiveness of the treatment and making adjustments to ensure that the patient’s needs are being met.
3. Assurance that each patient feels heard, understood and supported.
4. Provision of individual add-on interventions that address the potential unmet needs that cannot be fulfilled in the group context. Based on our findings, these include: needs for individual attention, psychological exploration, a focus on the patient’s life outside of therapy, extended assessment, and agreement on therapeutic tasks.

### Strengths and Limitations

This study concerns group CBT in Danish outpatient mental health services, which is coordinated into structured treatment programs. This might affect the transferability of results to other group psychotherapies such as interpersonal, psychodynamic, or mentalization-based, but also to CBT groups that are less standardized and time-restricted. However, the results might be relevant for other mental health services that provide standardized group therapy.

The interviews were performed retrospectively, which might imply that our findings would have been different if we had interviewed the patients while in therapy. However, the retrospective interviews may have allowed the participants to view the therapy courses as a whole and capture reflections that had appeared during the process. The relative flexibility and openness of the interview schedule seemed to allow for unexpected findings to emerge, in line with potentials in qualitative research.

The first author is a psychologist and conducted the interviews in the outpatient clinic. This could potentially have inhibited the patients’ willingness to talk freely about limitations of the treatment, since they may have perceived the interviewer as a part of the mental health service. However, the interviewer’s profession also contributed to a preunderstanding of psychological mechanisms and therapy processes that may have benefitted the interview process.

One of the therapists interviewed also participated in the research meeting where the themes of the study were discussed. While we perceive it as a strength to include clinicians in the analysis of findings, it must be noted as a limitation that no patients were involved in the analytic process.

We have only included patients and therapists from one mental health clinic, which may introduce a selection bias. However, the choice to only include one mental health clinic has the advantage that we are examining patients’ and therapists’ perspectives within the same structure, organizational culture, and population.

The current study makes a novel contribution to the field by focusing on standardized group CBT with ROM, underlining that ROM in group therapy might be insufficient for NOT patients if the feedback does not lead to intervention initiatives. This is in line with suggestions based on findings from individual therapy with ROM (Brattland et al., 2019; McAleavey & Moltu, 2021).

## Conclusion

The findings demonstrate that even within the same context, diagnosis, and form of therapy, patients have different needs and might potentially profit from a personalized approach. We suggest that patients who are persistently NOT in group psychotherapy benefit from a supplementary individual focus where the patient and therapist, through shared decision-making, decide how to personalize the treatment. In the current study, patients and therapists expressed different experiences and patient needs, indicating that personalizing the treatment will result in different types of intervention depending on the specific patient. These diverse needs, reflected in the main themes, provide a basis for content development for add-on interventions to group CBT that can be delivered in case of a lack of progress. In a future study, we will explore complementing group CBT with individual add-on interventions meeting patient needs for individual attention, psychological exploration, a focus on the patient’s life outside of therapy, extended assessment, and agreement on therapeutic tasks.

## Electronic Supplementary Material

Below is the link to the electronic supplementary material.

Supplementary Material 1
