# Supplementary material for: Patients’ and Therapists’ Experiences of Standardized Group Cognitive Behavioral Therapy: Needs for a Personalized Approach
Source: Adm Policy Ment Health. 2023 Sep 23;51(5):617–33. doi: 10.1007/s10488-023-01301-x (PMC11379796; doi:10.1007/s10488-023-01301-x)
Supplement: Supplementary file 1 — Supplementary Material 1 [file 10488_2023_1301_MOESM1_ESM.docx]

**Supplementary material: Interview schedules**

**Interview schedule – patients**

| **Purpose** | **Interview Questions** |
| --- | --- |
| **Examining the patient’s experience of the therapy course** | First, I would like you to tell me about the therapy course you have been in – just say what comes to your mind about it.  Can you describe your therapy course for me?  How do you understand your therapy course?  Can you describe the best and the worst about this therapy course?  What were your expectations for the therapy course?  What have you taken with you from the therapy?  Has the therapy changed you? (If yes), can you describe the change?  Can you give an example from your daily life of how the treatment has changed you?  What was helpful?  What was challenging?  What were you missing in the therapy?  Were the things you worked on relevant for you?  How was this therapy course in comparison to previous therapy you have tried? |
| **Examining the patient's experience with the therapists and group members** | How did you experience the therapists? Have you felt that you had problems the therapists failed to see or did not understand?  How was your experience with the other group members? |
| **Examining the patient's experience of her psychological state** | How do you feel today compared to before you started therapy?  How is it for you to feel that way?  Has something in your life happened that has affected you?  Which changes have you been through outside of therapy that affects your psychological state? What is your hope for the future?  What do you need now to feel better?  What have your friends and family suggested you to do now? |
| *Inclusion of illustrations of the patient’s ROM trajectories* | |
| **Examining the patient’s experience of seeing the trajectories** | We will now talk about the app and your trajectories.  Can you describe the trajectories for me?  How is it to see these trajectories?  How do the trajectories fit with your experiences?  Do you notice anything or remember something when looking at the trajectories? |
| **Examining the patient's experience of specific points on the trajectories** | Do you remember what happened here? (*e.g., where the curve goes down, where the curve goes up, or where the curve does not change)*  What was the reason that you dropped out of therapy here?/ What was the reason that you continued therapy? |
| **Examining the patient's needs when there was no progress** | If you were to have some additional treatment than group therapy, what would that be?  Why would that be helpful?  What made you feel that the treatment was helpful (if the patient experienced that)  How would it look if you could design the psychiatric/ therapeutic help? |
| **Examining specific add-on interventions** | Based on your and other patients' and therapists' experiences, we want to develop individual sessions that will be included when a group member is not progressing positively during therapy.  What do you think about that?  What could be helpful that the individual sessions concerned?  How would it be for you if one of the group therapists and you worked on a better understanding of you and your problems?  How would it be if you and the therapist went deeper into your history?  Does trying to solve some of the problems outside of therapy make sense?  Do you have other thoughts on what might be helpful? |
| **Examining the patient’s experience of using ROM with feedback** | How was it for you to reply to the questionnaires on the app?  How have you applied the app and the trajectories?  How did you experience that the therapists applied your replies to the questionnaires? |
| **Closing** | Is there anything I need to ask you about or that you would like to tell me? |

**Interview schedule - therapists**

| **Purpose** | **Interview Questions** |
| --- | --- |
| **Examining the therapist’s experience of the group** | First, I would like to know how you experienced this group?  How did you experience the patients’ relations to each other and their collaboration? |
| **Examining the therapist’s experience of the therapy course** | What did you experience that the patients found helpful?  What did you experience that the patients found challenging? What did you experience as challenging?  How was it to implement psychotherapeutic techniques in this group?  What were you missing to be able to offer in the treatment?  Do you think the patients were missing something? |
| **Examining the therapist's experience of the patients' psychological states** | Did any of the patients make a particular impression on you?  Which patients did you experience got something out of this, and which patients did you experience didn't get so much out of it? |
| **Examining the therapist's experience of the patients' psychological states** | Can you tell me about (*name of patient)*?  How was the therapy course of (*name of patient)*?  Did you experience something challenging about working with (*name of patient)*?  How do you experience that (*name of patient)* is feeling today compared to before the beginning of therapy?  How can you feel that (*name of patient)* is/ is not feeling better?  How is it for you to experience that (*name of patient)* is/ is not feeling better?  What does (*name of patient)* need now?  What were you missing to be able to offer in the treatment for (*name of patient)*?  How do you think (*name of patient)* experience the treatment? |
| *Inclusion of illustrations of patients’ ROM trajectories* | |
| **Examining the therapist’s experience of looking at the progress trajectories** | I would like to talk about (*name of patient)* again.  How is it for you to look at (*name of patient)*’s graphs? |
| *The Interviewer mentions two patients (extreme cases)* | |
| **Examining the therapist’s experience of specific points on the trajectories** | Do you remember what happened here? *(E.g., where the curve goes down, doesn't change or goes up)*.  How did you react when you experienced that (*name of patient) did not feel better or felt worse?*  What was the reason why (*name of patient)* dropped out of therapy here? / What was the reason that (*name of patient)* continued in therapy? |
| **Examining the patient’s need when there is no positive progress** | What was helpful when (*name of patient)* felt worse? What did you try to do? How did it work? Could other things have been beneficial?  What do you think contributed to (*name of patient)* feeling better?  How would it look if you could design the psychiatric/ therapeutic help for (*name of patient)*?  How do you think (*name of patient)* would have preferred the treatment? |
| **Examining specific add-on interventions** | Based on your and other therapists’ and patients' experiences, we would like to develop content for some individual sessions that will be added when a patient is not feeling better during therapy.  What would be helpful that these individual sessions contained?  Would it have been helpful for any of the patients if we added individual sessions where you worked on getting a better understanding of them?  Would it have been helpful if the individual sessions consisted of a thorough diagnostic assessment?  Would it have been helpful if the individual sessions consisted of concrete problem solving and, e.g., could contain calling a caseworker or something like that?  Do you have other thoughts on what could have been helpful? |
| **Examining the therapist’s experience of applying ROM with feedback** | How did you follow the patients' replies to the questionnaires?  How did you apply the replies to the questionnaires and the clinical support tool? |
| **Closing** | Is there anything I need to ask you or that you would like to tell me? |
